# Spatial Mapping of Bioactive Metabolites in the Roots of Three *Bupleurum* Species by Matrix-Assisted Laser Desorption/Ionization Mass Spectrometry Imaging

**DOI:** 10.3390/molecules29163746

**Published:** 2024-08-07

**Authors:** Xiaowei Han, Donglai Ma, Jiemin Wang, Lin Pei, Lingdi Liu, Weihong Shi, Zhengpu Rong, Xiaoyuan Wang, Ye Zhang, Yuguang Zheng, Huigai Sun

**Affiliations:** 1 College of Pharmacy, Hebei University of Chinese Medicine, Shijiazhuang 050200, China; hanxiaowei2015@126.com (X.H.); mdl_hebei@aliyun.com (D.M.); wjm15030331834@163.com (J.W.); s17333497047@163.com (W.S.); 18573787295@163.com (Z.R.); 19803318743@163.com (X.W.);; 2 Hebei Academy of Traditional Chinese Medicine, Shijiazhuang 050031, China; 13831190309@163.com; 3 Institute of Cash Crops, Hebei Academy of Agriculture and Forestry Sciences, Shijiazhuang 050051, China; nkyliulingdi@126.com

**Keywords:** *Bupleurum*, saikosaponins, non-saikosaponins, saikosaponin synthesis pathway compounds, MALDI-MSI, OPLS-DA, HPLC

## Abstract

*Bupleurum* is a kind of medicinal plant that has made a great contribution to human health because of the presence of bioactive metabolites: *Bupleurum* saikosaponins and flavonoids. Despite their importance, it has been a challenge to visually characterize the spatial distribution of these metabolites in situ within the plant tissue, which is essential for assessing the quality of *Bupleurum*. The development of a new technology to identify and evaluate the quality of medicinal plants is therefore necessary. Here, the spatial distribution and quality characteristics of metabolites of three *Bupleurum* species: *Bupleurum smithii* (BS), *Bupleurum marginatum* var. *stenophyllum* (BM), and *Bupleurum chinense* (BC) were characterized by Matrix-assisted laser desorption/ionization mass spectrometry imaging (MALDI-MSI). Twenty-nine metabolites, including saikosaponins, non-saikosaponins, and compounds from the saikosaponin synthesis pathway, were characterized. Some of these were successfully localized and visualized in the transverse section of roots. In these *Bupleurum* species, twelve saikosaponins, five non-saikosaponins, and five saikosaponin synthesis pathway compounds were detected. Twenty-two major influencing components, which exhibit higher ion intensities in higher quality samples, were identified as potential quality markers of *Bupleurum*. The final outcome indicates that BC has superior quality compared to BS and BM. MALDI-MSI has effectively distinguished the quality of these *Bupleurum* species, providing an intuitive and effective marker for the quality control of medicinal plants.

## 1. Introduction

*Bupleurum* is a genus of the Apiaceae, comprising about 200 species. In China, about 44 species, 17 varieties, and 7 forma have been identified and are distributed widely across the country in 27 provinces [1,2]. Chaihu (Bupleuri Radix), the dried roots of *Bupleurum chinense* DC. and *B. scorzonerifolium* willd., is a famous Traditional Chinese medicinal herb with a history of medical use for more than 2000 years. Its main bioactive metabolites are saikosaponins and flavonoids. Saikosaponins and flavonoids are the main effective components of *Bupleurum*, and they can help treat diseases [3]. To measure the quality of *Bupleurum*, HPLC and GC-MS can be used to identify saikosaponins and flavonoids in *Bupleurum* [4,5]. This traditional method for the identification of the quality of *Bupleurum* could not be used to detect the spatial distribution of saikosaponins or other components in *Bupleurum*.

The spatial distribution of the main effective components of medicinal plants is important information regarding medicinal plants. Studies on the spatial distribution of saikosaponins and other effective components in *Bupleurum* provide direct information on the state of different growth stages of *Bupleurum*. The information can help with the study of the physiological and biochemical characteristics of saikosaponins and develop drugs based on saikosaponins.

MALDI-MSI technology has the ability to obtain specific images of metabolite molecules, without special labeling and staining, which is very convenient and fast. It has become an important technology to characterize the spatial characteristics of active components of medicinal plants, and has shown great potential in many research fields [6,7,8]. MALDI-MSI has made important contributions in many fields of biological science and has become a new research direction of plant metabonomics [9,10]. The main function of MALDI-MSI is to capture various chemical components in the space of biological tissues [11,12]. MALDI-MSI technology is constantly being updated and developed, including the development of MS instruments with higher resolution and higher sensitivity. These technologies make proteins, lipids, and various secondary metabolites in biological tissues and cells show specific spatial and temporal changes [13,14,15].

The achievements of MALDI-MSI technology in the research of medicinal plant pharmacological components have made it possible to use this technology to study the synthesis pathway of saikosaponins. MALDI-MSI imaging can be used to study the content and space distribution of maytansinoids in different plant species of Celastraceae to expand the source of maytansinoids [16]. MALDI-MSI was used to understand the space distribution of xanthones and bifidus flavonoids in thin sections of *G. biloba* leaves. The results indicate that *G. biloba* leaves also contain pharmacodynamic components to be used as drugs [17]. High-resolution MALDI mass spectrometry imaging of gallic acid and monoterpene glycosides in *Paeonia lactiflora* and *Glycyrrhiza uralensis* roots provides important clues for understanding the biosynthetic pathway of gallic acid and monoterpene glycosides [18]. The spatiotemporal localization of flavonoid metabolites in strawberry fruits revealed that flavonoids only existed in red fruits, thus indicating that mature fruits were more beneficial to people’s health [19].

Here, we researched the distribution of bioactive metabolites in three *Bupleurum* roots by MALDI-MSI. *Bupleurum chinense* (BC) is a typical medicinal plant that can soothe the liver, regulate qi, activate blood circulation, and relieve pain. Clinically, it is mainly used to treat depression, Alzheimer’s disease, functional dyspepsia, and cardiovascular and gynecological diseases [20]. The bioactive metabolites of *Bupleurum* contain saikosaponins, polysaccharides, total flavonoids, volatile oils, and other components, but saikosaponins are the most prevalent and the main component of its efficacy [21]. *Bupleurum* is widely planted in China. There are many species in the genus *Bupleurum*, and different contents of pharmacodynamic components in *Bupleurum* lead to large differences in their pharmacodynamics [22]. Although the difference in components of various *Bupleurum* species has been studied, the spatial distribution of these components in the secondary metabolism process has not yet been elucidated. Here, three species of the genus *Bupleurum* were used to measure the differences in their components and the spatial distribution of the main pharmacodynamic components including saikosaponins A, B, C, and D.

## 2. Results

### 2.1. Detection of Saikosaponins, Non-Saikosaponins, and Saikosaponin Synthesis Pathway Compounds by MALDI-TOF MS

**Figure 1:**
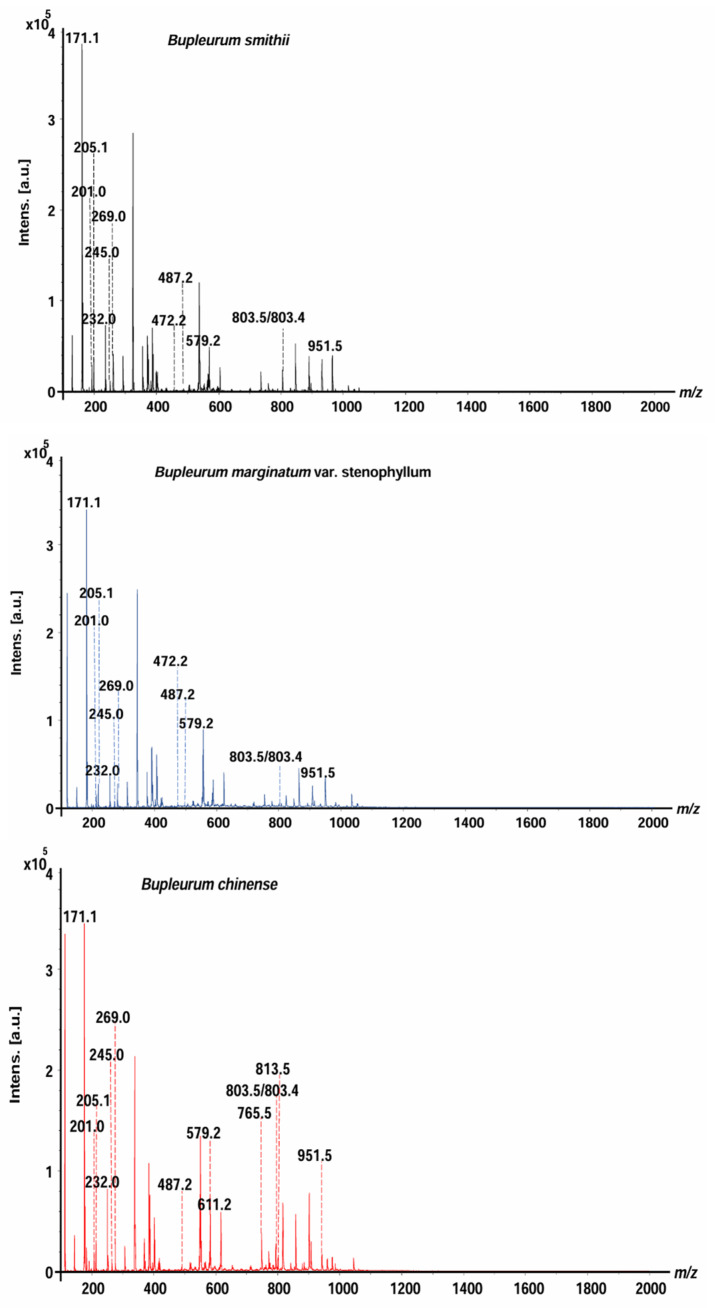
The overall average mass spectra of the three *Bupleurum* species obtained by MALDI-MS.

*B. smithii* (BS), *B. marginatum* var. *stenophyllum* (BM), and BC were analyzed by MALDI-MS (Figure 1). The mass spectra of saikosaponins, saikosaponin synthesis pathway compounds, and non-saikosaponin components exhibit distinct characteristics across the three species of *Bupleurum*, offering compelling evidence for their identification and differentiation. The saikosaponins, non-saikosaponins, and saikosaponin synthesis pathway compounds of the three *Bupleurum* roots were tentatively determined based on their molecular weight measurements, MS/MS fragmentation patterns, and characteristic fragment ions. There were 12, 12, and 14 peaks for BS, BM, and BC, respectively. These were identified as saikosaponins, non-saikosaponins, and saikosaponin synthesis pathway compounds (Table 1). The detected ions were prominently in the potassium, sodium, and hydrogen adduct forms of saikosaponins, non-saikosaponins, and saikosaponin synthesis pathway compounds. Some common peaks were identified as saikosaponin A (*m*/*z* 951.5), saikosaponin B3 (*m*/*z* 813.5), saikosaponin C (*m*/*z* 765.5), saikosaponin E/F (*m*/*z* 803.4), saikosaponin F/E (*m*/*z* 803.5), non-saikosaponin (*m*/*z* 611.2, *m*/*z* 579.2, *m*/*z* 487.2, *m*/*z* 245.0, and *m*/*z* 205.1), and saikosaponin synthesis pathway compounds (*m*/*z* 472.2, *m*/*z* 269.0, *m*/*z* 232.0, *m*/*z* 201.0, and *m*/*z* 171.1).

### 2.2. Statistical Analysis of Variables for Saikosaponins, Non-Saikosaponins, and Saikosaponin Synthesis Pathway Compounds in the Three Bupleurum Species

**Figure 2:**
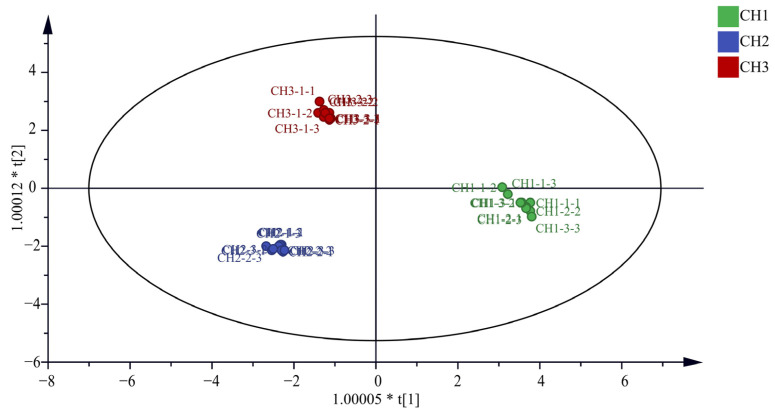
Plot of OPLS-DA model scores for the three *Bupleurum* species.

It can be seen from Figure 2 that the OPLS-DA model distinguished the three *Bupleurum* samples. In addition, this study also conducted the OPLS-DA model verification, in which the replacement test *n* = 200. The results show that R^2^ = 0.0111, Q^2^ = −0.411, R^2^ and Q^2^ on the right are higher than those on the left, and Q^2^ intersects with the Y-axis on the negative axis, indicating that the model is reliable and does not have overfitting phenomenon (Supplementary Materials Figure S1). At the same time, the VIP value is used as the criterion for screening differential metabolites. If the VIP value is greater than 1, it indicates that this compound can be used as the differential component of the three samples. It can be seen from the Supplementary Materials Figure S2 that the VIP values of saikosaponin C (*m*/*z* 765.5), saikosaponin E/F (*m*/*z* 803.4), saikochromic acid (*m*/*z* 245.0), dimethoxy-isoflavane-7-o-β-D-glucoside (*m*/*z* 487.2), (R)-mevalonic acid (*m*/*z* 171.1), isopentenyl pyrophosphate (*m*/*z* 269.0), mevalonate-5-phosphate (*m*/*z* 201.0), tryptophan (*m*/*z* 205.1), and mevalonate-5-pyrophosphate (*m*/*z* 232.0) are greater than 1. The VIP values are as follows: 1.22, 1.20, 1.17, 1.14, 1.07, 1.07, 1.07, 1.06, and 1.06, respectively.

### 2.3. Determination of Saponins in Bupleurum by HPLC

**Figure 3:**
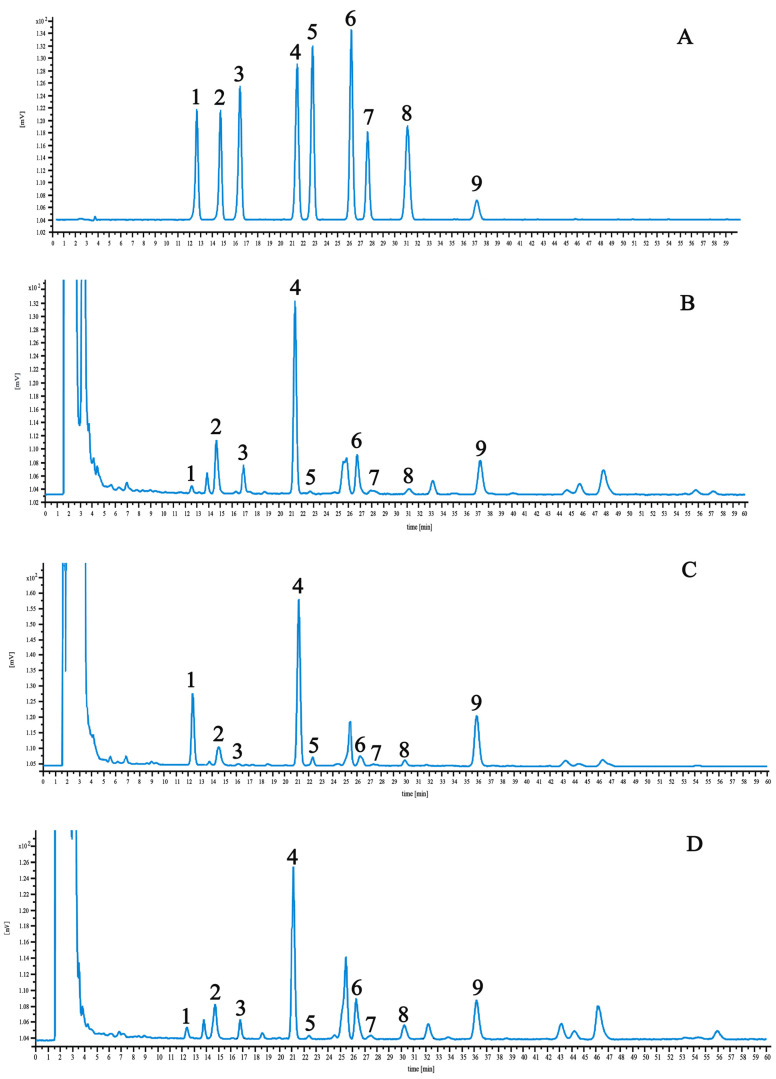
HPLC chromatograms of reference compound (**A**), BS (**B**), BM (**C**), and BC (**D**). 1—saikosaponin C; 2—saikosaponin F; 3—saikosaponin B3; 4—saikosaponin A; 5—saikosaponin B2; 6—saikosaponin G; 7—saikosaponin B1; 8—saikosaponin E; 9—saikosaponin D.

The linear regression data and results are detailed in Table 2. Optimized chromatographic conditions ensured the clear resolution of these components, as shown in the chromatograms of control and test samples presented in Figure 3. Among the nine components, the highest saikosaponin A content was found in BS and BM, which have 2.84 and 2.80 mg·g^−1^, respectively. BM had the highest saikosaponin C content at 3.21 mg·g^−1^. BS and BC had the lowest saikosaponin B2 content of 0.1 and 0.13 mg·g^−1^, respectively.

It can be seen from the experimental results that there are degrees of differences in the content of the measured components in the *B. radix* samples from different origins, the content of saikosaponin B3 in different sources is relatively large, and the difference between saikosaponin B1 and saikosaponin E is relatively small. This shows that the difference in the content of saikosaponin B3 is closely related to the place of origin (Table 3).

### 2.4. Localization of Saikosaponin in the Root Tissues of the Three Bupleurum Species

**Figure 4:**
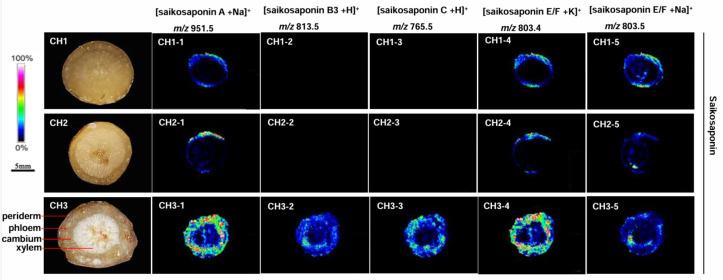
The localization of saikosaponin ions in the root tissues of the three *Bupleurum* species by MALDI-MSI. CH1: BS; CH2: BM; and CH3: BC.

The mass spectrometry data showed that the chemical components contained in the three *Bupleurum* species were similar (Figure 4). This indicates that the pharmacodynamic components of these species are comparable. However, the targeted results showed significant differences in the composition and content of some compounds. There are 12 kinds of saikosaponins in BC: saikosaponin A, B1, B2, B3, B4, C, D, E, F, G, H, and I. Similar mass-to-charge ratios were found in some groups of saikosaponins, e.g., saikosaponins A, B1, B2, and D; B3 and B4; F, H, and I; E and G. The mass-to-charge ratio of saikosaponin C differed from the others. Twelve saikosaponins distributed in the periderm, phloem, and xylem of roots were detected in BC at high concentration. Only saikosaponins A, E, and F were detected in BM. Saikosaponin B3 and C were not observed in the root of BM. The MALDI-MSI results of BS were consistent with those of BM, only saikosaponins A, E, and F were detected at low abundance.

### 2.5. Localization of Saikosaponin Synthesis Pathway Compounds in the Three Bupleurum Root Tissues

**Figure 5:**
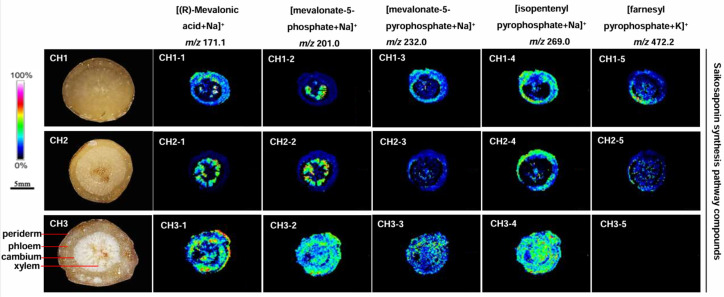
The localization of saikosaponin synthesis pathway compound ions in the root tissues of the three *Bupleurum* species by MALDI-MSI. CH1: *BS*; CH2: BM; and CH3: BC.

The content of saikosaponin synthesis pathway compounds in BC was abundantly distributed in the periderm, phloem, and xylem of roots (Figure 5). Four compounds-(R)-mevalonic acid, mevalonate-5-phosphate, mevalonate-5-diphosphate and isopentenyl pyrophosphate-were detected in BC, but farnesyl pyrophosphate was not observed. The five compounds were detected in BS and BM, but the content was significantly lower than in BC.

### 2.6. Localization of Non-Saikosaponin Compounds in the Three Bupleurum Root Tissues

**Figure 6:**
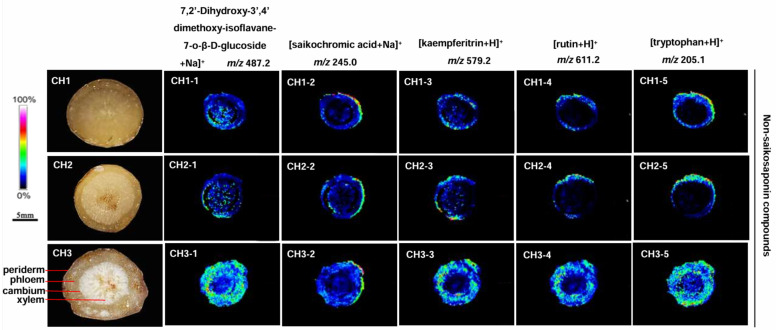
The localization of non-saikosaponin ions in the root tissues of the three *Bupleurum* species by MALDI-MSI. CH1: BS; CH2: BM; and CH3: BC.

Five non-saikosaponin compounds were detected, including 7,2′-dihydroxy-3′,4′-dimethoxy-isoflavane-7-o-β-D-glucoside, saikochromic acid, kaempferitrin, rutin, and tryptophan in BS, BM, and BC, but the content differed (Figure 6). The most abundant non-saikosaponin was observed in all parts of BC roots, while the content of BM and BS was significantly reduced and only seen in the cortex near the periderm. There was almost none in the phloem and xylem.

### 2.7. Cluster Analysis of Different Components

**Figure 7:**
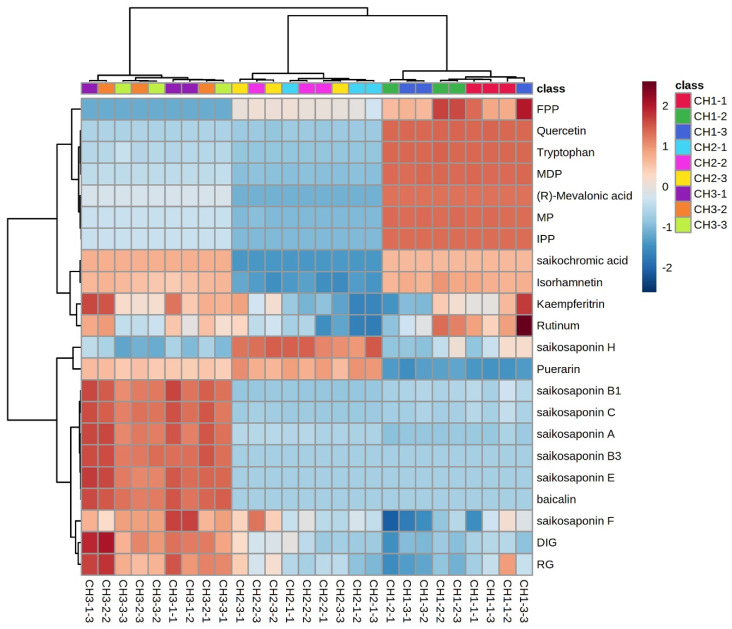
Cluster heatmap of chemical constituents of the three *Bupleurum* species. FPP: farnesyl pyrophosphate; MDP: mevalonate-5-diphosphate; MP: mevalonate-5-phosphate; IPP: isopentenyl pyrophosphate; RG: isorhamnetin-3-O-glucoside; and DIG: 7,2′-dihydroxy-3′,4′-dimethoxy-isoflavane-7-o-β-D-glucoside. CH1: BS; CH2: BM; CH3: BC.

The expression of the same compound in different *Bupleurum* species and different compounds in the same species are shown in Figure 7. We used the average value of the compound expression of the same *Bupleurum* sample as a benchmark: expression above the average value is positive and marked in red. Expression below the average is negative and marked in blue. The color depth indicates the difference between the expression amount of the compound and the average value. The expression of saikosaponins A, B1, B3, C, E, and F in BC was significantly higher than the average value, while the expression of saikosaponins in BS and BM was lower than the average value. However, the saikosaponin synthesis pathway compounds FPP, quercetin, tryptophan, MDP, (R)-mevalonic acid, MP and IPP had higher expression in BS and lower expression in BM and BC. Only the expression of puerarin and saikosaponin H was higher than the average expression in BM. Saikonchromic acid, isorhamnetin, kaempferitrin, and rutinum were lightly expressed in BC and BS, and were expressed less in BM. The saikosaponin content is the most important indicator to assess the quality of *B. radix*; thus, the quality of BC is better than that of BS and BM.

## 3. Discussion

The main effective component of *B. radix* is *Bupleurum* saikosaponin [21]. Twelve *Bupleurum* saikosaponins were reported, namely saikosaponin A, B1, B2, B3, B4, C, D, E, F, G, H, and I [4], (Supplementary Materials Figure S3). Saikosaponins have different pharmacological effects. Saikosaponin A has anti-human neuroblastoma effects and limits bladder cancer cell growth [20,23]. Saikosaponins A and D can inhibit lipogenesis [24]. Saikosaponins A, B, and D can restrict the growth of transplanted mouse medulloblastoma [25]. Saikosaponins F and G also have different anti-cancer effects [26].

*Bupleurum* saikosaponin is the main active ingredient and an important secondary metabolite of *B. radix*. The MALDI analysis showed variations in the distribution of saikosaponin in different *Bupleurum* species. BC contains all types of *Bupleurum* saponins (Figure 4; *m*/*z* 803.4, *m*/*z* 813.5, *m*/*z* 803.5, *m*/*z* 765.5, and *m*/*z* 951.5) in all areas of roots. BM does not contain *Bupleurum* saikosaponins B and C (Figure 4; *m*/*z* 813.5 and *m*/*z* 765.5); there is only a trace amount of the other saikosaponins near the periderm of the root and almost none in the xylem. The distribution and content of saikosaponins in BS and BM were similar. The quality of BS is the best among the three species in terms of saikosaponin content.

Saikosaponins are secondary metabolites of *Bupleurum*. Transcriptomic and metabolomics studies have found some key enzymes and compounds in the synthetic pathway of saikosaponin [27,28,29]. MALDI was used to characterize some compounds in the saikosaponin synthesis pathway of *Bupleurum* roots (Supplementary Materials Figure S4). This could nicely display the position and content of these compounds in *Bupleurum* roots. Here, (R)-mevalonic acid was mainly distributed in the periderm and phloem of BS and the periderm, phloem, and xylem of BC; it was only seen in the xylem of BM (Figure 5, *m*/*z* 171.1). Mevalonate-5-phosphate is only distributed in the xylem of BS and BM. The entire root area of BC has high levels of mevalonate-5-phosphate (Figure 5, *m*/*z* 201.0). Mevalonate-5-pyrophosphate and isopentenyl pyrophosphate are mainly distributed in the periderm and phloem of BS and BM as well as in the entire root of BC (Figure 5; *m*/*z* 232.0, *m*/*z* 269.0). However, it is puzzling that farnesyl pyrophosphate is distributed in the whole root of BS and BM despite its low content. Farnesyl pyrophosphate is not detected in BC (Figure 5; *m*/*z* 472.2). A possible explanation is that BC has not yet formed farnesyl pyrophosphate, which also indicates the time difference in saikosaponin for the formation of these *Bupleurum* species. The analysis of the key compounds of the saikosaponin synthesis pathway also showed that BC had the best quality of saikosaponin.

**Figure 8:**
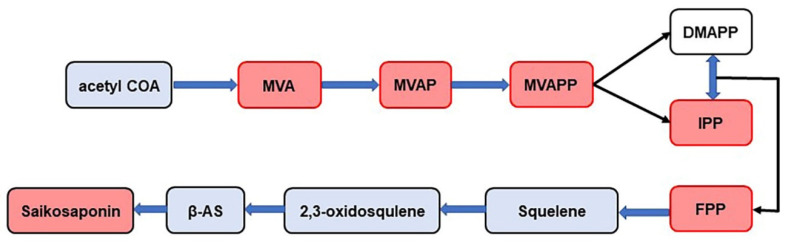
Synthetic pathway of saikosaponin (the compounds in the red box are the compounds identified in this experiment). MVA: mevalonic acid, MVAP: mevalonate-5-phosphate, MVAPP: mevalonate-5-pyrophosphate, DMAPP: dimethylallyl diphosphate, IPP: isopentenyl diphosphate, FPP: farnesyl pyrophosphate, β-AS: β-amyrin.

Saikosaponins are triterpenoid saponins of the oleanane type. The pathway of their synthesis has been studied in detail (Figure 8). This pathway is called the MVA pathway. MVA is an important site in the metabolism of cytoplasmic terpenoids. MVA forms IPP and DMAPP through a series of reactions. They are precursors of all terpenoids and steroids. IPP and DMAPP further formed FPP under the catalysis of isopentenyl transferase, and FPP finally produced triterpene saponins through a series of downstream reactions. In this study, MVA, MVAP, MVAPP, IPP, and FPP were detected in BS, BM, and BC by MALDI-MS technology (Figure 8), indicating that the three *Bupleurum* species have the same synthetic process of saikosaponin, but the amount of products are different. The content of the saikosaponin synthesis pathway compounds indicates the final quantity of saikosaponin, which provides a basis for determining the quality of *Bupleurum*. Meanwhile, the results also revealed that certain compounds in the synthetic pathway were not detected, especially in the FPP pathway, e.g., beta-AS, 2,3-oxidosqualene and squalene. It was speculated that the reason for this might be the incompatibility of the chemical structures of 2-MBT with individual compounds in the FPP pathway, which resulted in the inability of these compounds to be efficiently ionized or to produce characteristic signals in the mass spectra.

There are a few non-saikosaponins in *Bupleurum* including 7,2′-dihydroxy-3′,4′-dimethoxy-isoflavane-7-o-β-D-glucoside, *Bupleurum* xanthan ketonic acid, kaempferitrin, baicalin, rutin, tryptophan, quercetin, isorhamnetin 3-o-glucoside, isorhamnetin, and puerarin (Supplementary Materials Figure S5) [30]. Five non-saikosaponin components were identified in BS, BM, and BC by the MALDI-MSI technology including 7,2′-dihydroxy-3′,4′-dimethoxy-isoflavane-7-o-β-D-glucoside (Figure 6, *m*/*z* 487.2), saikochromic acid (Figure 6, *m*/*z* 245.0), kaempferitrin *m*/*z* 579.2), rutin (Figure 6, *m*/*z* 611.2), and tryptophan (Figure 6, *m*/*z* 205.1). In BS, BM, and BC, these components are all located in the periderm and phloem of roots with a trace abundance in the xylem. The content of these components in BC was significantly higher than that in BS and BM. Although the non-saikosaponin components are not the main pharmacodynamic components of BC, they still have certain pharmacological effects. In conclusion, the quality of BC is better than that of BS and BM.

## 4. Materials and Methods

### 4.1. Materials

Seeds of *Bupleurum smithii* (BS), *B. marginatum* var. *stenophyllum* (BM), and *B. chinense* (BC) were purchased from Inner Mongolia, Gansu, and Hebei Provinces, respectively. They were planted in the Medicinal Plant Resource Garden of Hebei Academy of Agricultural and Forestry Sciences, Shijiazhuang, Hebei province, China (38.04228 N 114.5144 E) on August 2021. Each *Bupleurum* species was planted in a single plot: three rows that were 3 m long with 1.5 m between rows. Thirty seeds were sown in each row. After growing for 1 year, the roots were collected for MALDS-MSI in July 2022. Three seedlings were taken from each row of a community, and thus nine seedlings were sampled from each *Bupleurum* species.

Saikosaponin standards were purchased from Shanghai Yuanye Bio-Technology Co., Ltd., Shanghai, China (www.shyuanye.com, accessed on 3 April 2024). Detailed information on the standards is provided in the Supplementary Materials.

### 4.2. Tissue Sectioning

Clean the fresh *Bupleurum* roots, remove the fibrous roots, and cut them transversely into segments about 0.5 cm in length. Embed the freshly cut tissue with OCT (Optimal Cutting Temperature) freezing section embedding agent on the sample tray of the cryostat, and place it on the ultra-low temperature freezing stage (−20 °C, Leica Microsystems Inc., Wetzlar, Germany) inside the cryostat for 10 min. Cut into 12 μm thick adjacent sections of *Bupleurum* root tissue, then transfer them onto ITO (indium tin oxide) coated conductive glass slides (Bruker Daltonics, Billerica, MA, USA), and vacuum dry for 20 min.

### 4.3. Matrix Coating and Microscopy Visualization

A 10 mg/mL solution of 2-mercaptobenzothiazole (2-MBT) was formulated using a methanol-to-water solvent blend at an 80:20 ratio by volume, supplemented with 0.2% trifluoroacetic acid (TFA). The application of this matrix to the tissue sections of *Bupleurum* was executed with an automated spotter, the ImagePrep, from Bruker Daltonics, in Bremen, Germany. To achieve a consistent and thin layer of matrix, a continuous spray application was administered for a duration of 5 s, succeeded by a 60 s interval for evaporation. This process was iterated five times. Post-initial cycles and after natural air drying in a ventilated area, another 40 cycles of even matrix spraying were applied to the tissue sections.

### 4.4. MALDI-MSI

The instrument used for all profile and imaging experiments is a Bruker Autoflex Speed MALDI time-of-flight (TOF)/TOF mass spectrometer (Bruker Daltonics). The mass range of all mass spectra is *m*/*z* 100 to 3500. Use 20 laser scanning accumulations to record the mass spectrum to obtain the profile data of MALDI-MS. For imaging data acquisition, endogenous low-MW compounds in the *Bupleurum* root tissue sections were detected by 100 μm laser raster step-sizes. The compounds used for external mass calibration and their *m*/*z* value: Pro ([M + H]^+^, *m*/*z* 116.1), bradykinin 1−7 ([M + H]^+^, *m*/*z* 757.4), angiotensin II ([M + H]^+^, *m*/*z* 1046.5), angiotensin I ([M + H]^+^, *m*/*z* 1296.7), substance P ([M + H]^+^, *m*/*z* 1347.7), and bombesin ([M + H]^+^, *m*/*z* 1619.8). Matrix ions of DMCA ([M + H]^+^, *m*/*z* 209.1) and 2-MBT ([M + H]^+^, *m*/*z* 168.0) were used for an internal mass calibration. The calibration is the cubic-enhanced mode.

### 4.5. Data Analysis

Profiling data of saikosaponin, non-saikosaponin, and saikosaponin synthesis pathway compounds were checked and treated by the Bruker FlexAnalysis 3.4 software (https://softwaretopic.informer.com/, accessed on 22 April 2024). The mass window was 0.3% and the signal-to-noise (S/N) ratio was 3. The ion maps of bioactive metabolites of *Bupleurum* root tissues were processed by The Bruker FlexImaging 4.1 software (https://sourceforge.net/, accessed on 22 April 2024). The 3D maps were generated by the PD Quest 2-D Analysis 8.0.1 software (Bio-Rad, Hercules, CA, USA).

### 4.6. Histological Staining

After the MALDI-MS experiment, the matrix on all tissue sections was cleaned with methanol solution of three concentrations (70%, 90%, and 100%), and then stained with hematoxylin and eosin. The results of the H&E staining are shown in the Supplementary Materials Figure S6.

### 4.7. Preparation of Standard Compounds

Standard solutions of 12 saikosaponins, 10 non-saikosaponins, and 8 saikosaponin synthesis pathway compounds were prepared for MALDI-TOF MS detection. Details of the standards are given in the Supplementary Materials Figures S3–S5.

### 4.8. Extraction and Content Determination of Saikosaponins by HPLC

The saponins were extracted from *Bupleurum* roots using HPLC and their contents were determined. The detailed extraction process and chromatographic conditions are recorded in the Supplementary Materials.

## 5. Conclusions

The evaluation of medicinal plants is inherently challenging, with the production of efficacious components being influenced by cultivation methods and plant species. While HPLC and GC-MS methods have been employed to quantify the content of various components in *B. radix*, differentiating between varieties of *B. radix* remains non-intuitive. However, MALDI-MSI offers a visual characterization of the differences between the three *Bupleurum* species, including BC, which has been found to be superior in quality to BS and BM. This technique has been used to detect twelve saikosaponins, five non-saikosaponins, and five saikosaponin synthesis pathway compounds in these species. By analyzing the quality and the spatial distribution of pharmacodynamic substances, MALDI-MSI provides a direct observation and calibration method for the effective components of medicinal plants.

## Figures and Tables

**Table 1:** Saikosaponin, non-saikosaponin, and saikosaponin synthesis pathway compounds detected in the three *Bupleurum* species by MALDI-TOF-MSI and confirmed by MALDI-TOF-MS/MS.

Measured (*m*/*z*)	Calculated (*m*/*z*)	Error (ppm)	Identification			Localization			CID Fragment Ions
			Ion Form	Compound	Molecular Formula	BS	BM	BC	
951.5	951.5287	2	[M + Na]^+^	Saikosaponin A	C_48_H_80_O_17_	Periderm	Periderm	Periderm and phloem	365.1; 451.1; 511.2; 805.5; 919.3
803.4	803.4342	3	[M + K]^+^	Saikosaponin E/F	C_42_H_68_O_12_	Periderm	Periderm	Periderm, phloem, and cambium	203.1; 331.2; 349.1; 757.4
813.5	813.4994	2	[M + H]^+^	Saikosaponin B3	C_43_H_72_O_14_	-	-	Periderm, phloem, and cambium	133.1; 251.1; 309.1; 559.4; 705.5
803.5	803.4552	1	[M + Na]^+^	Saikosaponin E/F	C_42_H_68_O_13_	Periderm	Periderm	Periderm, phloem, and cambium	455.4; 437.3; 456.3
765.5	765.4783	1	[M + H]^+^	Saikosaponin C	C_48_H_78_O_18_	-	-	Periderm, phloem, and cambium	189.2; 255.1; 403.3; 649.9
611.2	611.1606	1	[M + H]^+^	Rutin	C_27_H_30_O_16_	-	-	Periderm, phloem, and cambium	303.0; 611.0; 612.0
579.2	579.1708	4	[M + H]^+^	Kaempferitrin	C_27_H_30_O_14_	Periderm, phloem, and cambium	Periderm, phloem, and cambium	Periderm, phloem, and cambium	
487.2	487.24	1	[M + Na]^+^	7,2′-Dihydroxy-3′,4′-dimethoxy-isoflavane-7-o-β-D-glucoside	C_23_H_28_O_10_	Periderm, phloem, cambium, and xylem	Periderm, phloem, cambium, and xylem	Periderm, phloem, cambium, and xylem	
472.2	472.1738	7	[M + K]^+^	Farnesyl pyrophosphate	C_15_H_37_N_3_O_7_P_2_	Periderm, phloem, cambium, and xylem	Periderm, phloem, cambium, and xylem	-	160.9; 365.1; 383.2
269.0	268.9950	4	[M + Na]^+^	Isopentenyl pyrophosphate	C_5_H_12_O_7_P_2_	Periderm, phloem, and cambium	Periderm, phloem, and cambium	Periderm, phloem, cambium, and xylem	161.1; 229.0
245.0	245.06	2	[M + Na]^+^	Saikochromic acid	C_10_H_6_O_6_	Periderm, cambium, and xylem	Periderm, cambium, and xylem	Periderm, phloem, and cambium	
232.0	232.05	2	[M + Na]^+^	Mevalonate-5-pyrophosphate	C_6_H_12_O_2_P_2_	Periderm, phloem, and cambium	Periderm, phloem, and cambium	Periderm, phloem, cambium, and xylem	
205.1	205.11	4	[M + H]^+^	Tryptophan	C_11_H_12_N_2_O_2_	Periderm	Periderm	Periderm, phloem, cambium, and xylem	143.9; 145.9; 151.0; 187.9; 204.9
201.0	201.07	3	[M + Na]^+^	Mevalonate-5-phosphate	C_6_H_12_O_2_P	Xylem	Xylem	Periderm, phloem, cambium, and xylem	
171.1	171.0627	2	[M + Na]^+^	(R)-Mevalonic acid	C_6_H_12_O_2_	Periderm and phloem	Xylem	Periderm, phloem, cambium, and xylem	108.0; 149.7

**Table 2:** Regression data of nine saponins.

Analyte	Linear Range/(mg·mL^−1^)	Regression Equation	*r*
Saikosaponin C	0.21~1.11	Y = 1198.8X − 25.776	0.9985
Saikosaponin F	0.18~0.96	Y = 1145.5X + 26.776	0.9998
Saikosaponin B3	0.27~0.94	Y = 1214.1X − 5.034	0.9949
Saikosaponin A	0.22~1.17	Y = 1756.2X − 0.239	0.9990
Saikosaponin B2	0.26~1.35	Y = 1652.9X − 7.000	0.9999
Saikosaponin G	0.22~1.17	Y = 2223.2X − 41.657	0.9990
Saikosaponin B1	0.19~1.02	Y = 1341.6X − 43.723	0.9994
Saikosaponin E	0.19~0.99	Y = 1831.8X − 38.310	0.9990
Saikosaponin D	0.14~0.72	Y = 742.61X − 37.835	0.9991

**Table 3:** Sample determination result (mg·g^−1^).

Sample	Saikosaponin C	S. F	S. B3	S. A	S. B2	S. G	S. B1	S. E	S. D
BS	0.34 ^b^ ± 0.03	1.03 ^a^ ± 0.06	0.55 ^a^ ± 0.03	2.84 ^a^ ± 0.11	0.10 ^b^ ± 0.01	0.77 ^a^ ± 0.02	0.40 ^a^ ± 0.03	0.26 ^a^ ± 0.04	2.36 ^a^ ± 0.15
BM	3.21 ^a^ ± 0.12	1.05 ^a^ ± 0.06	0.12 ^c^ ± 0.01	2.80 ^a^ ± 0.08	0.29 ^a^ ± 0.03	0.54 ^b^ ± 0.03	0.38 ^a^ ± 0.02	0.32 ^a^ ± 0.04	2.49 ^a^ ± 0.14
BC	0.38 ^b^ ± 0.04	0.55 ^b^ ± 0.02	0.32 ^b^ ± 0.04	2.08 ^b^ ± 0.08	0.13 ^b^ ± 0.01	0.68 ^a^ ± 0.03	0.42 ^a^ ± 0.03	0.37 ^a^ ± 0.02	1.38 ^b^ ± 0.09

Note: This table presents the mean values and standard deviations. Mean values with different letters in the same column indicate statistically significant differences (*p* < 0.05).

## Data Availability

The data presented in this study are available in the article and Supplementary Materials.

## Supplementary Materials

The following supporting information can be downloaded at: https://www.mdpi.com/article/10.3390/molecules29163746/s1. Figure S1: The chemical structure of 12 saikosaponins in *Bupleurum*. Figure S2: The chemical structure of eight compounds of saikosaponin synthesis pathway in *Bupleurum*. Figure S3: The chemical structure of non-saikosaponin in *Bupleurum*. Figure S4: HE staining of three *Bupleurum*, CH1: *Bupleurum smithii*; CH2: *Bupleurum marginatum* var. *stenophyllum*; CH3: *Bupleurum chinense*. Figure S5: The chemical structure of non-saikosaponin in *Bupleurum*. Figure S6: HE staining of three *Bupleurum*. CH1: *Bupleurum smithii*; CH2: *Bupleurum marginatum* var. *stenophyllum*; CH3: *Bupleurum chinense*.

## References

[1] Yao R.Y., Zou Y.F., Chen X.F. (2013). Traditional use, pharmacology, toxicology, and quality control of species in genus *Bupleurum* L.. Chin. Her. Med..

[2] Yu D., Wang W.X., Huo J.H., Zhuang Y., Chen Y.Y., Du X.W. (2023). Study on molecular mechanism of volatiles variation during *Bupleurum scorzonerifolium* root development based on metabolome and transcriptome analysis. Front. Plant Sci..

[3] Chinese Pharmacopeia Commission (2020). Pharmacopoeia of the People’s Republic of China.

[4] Liu Y.M., Zhou A., Yu N.J., Han R.C., Zhang W., Zhu Y.J., Cao Y., Li X.Y., Peng D.Y. (2018). Simultaneous determination of five saponins in Bupleuri Radix by HPLC-DAD dual wavelength method. China J. Chin. Mat. Med..

[5] Deng P., Zheng G.S., Luo X.J., Wang Y.S. (2013). Simultaneous determination of saikosaponins a, b2, c, d, f in *Radix Bupleuri* by HPLC. J. Jiangxi Univ..

[6] Wu C.P., Dill A.L., Eberlin L.S., Cooks R.G., Ifa D.R. (2013). Mass spectrometry imaging under ambient conditions. Mass Spectrom. Rev..

[7] Dong Y.H., Li B., Malitsky S., Rogachev I., Aharoni A., Kaftan F., Svatoš A., Franceschi P. (2016). Sample preparation for mass spectrometry imaging of plant tissues: A review. Front. Plant Sci..

[8] Van Hove E.R.A., Smith D.F., Heeren R.M.A. (2010). A concise review of mass spectrometry imaging. J. Chromatogr. A..

[9] Heyman H.M., Dubery I.A. (2016). The potential of mass spectrometry imaging in plant metabolomics: A review. Phytochem. Rev..

[10] Kaspar S., Peukert M., Svatos A., Matros A., Mock H.P. (2011). MALDI-imaging mass spectrometry—An emerging technique in plant biology. Proteomics.

[11] Cornett D.S., Reyzer M.L., Chaurand P., Caprioli R.M. (2007). MALDI imaging mass spectrometry: Molecular snapshots of biochemicalsystems. Nat. Methods.

[12] Lee Y.J., Perdian D.C., Song Z., Yeung E.S., Nikolau B.J. (2012). Use of mass-spectrometry for imaging metabolites in plants. Plant J..

[13] Kompauer M., Heiles S., Spengler B. (2017). Atmospheric pressure MALDI mass spectrometry imaging of tissues and cells at 1.4-μm lateral resolution. Nat. Methods.

[14] Baker T.C., Han J., Borchers C.H. (2017). Recent advancements in matrix-assisted laser desorption/ionization mass spectrometry imaging. Curr. Opin. Biotechnol..

[15] Schwamborn K., Caprioli R.M. (2010). Molecular imaging by mass spectrometry—Looking beyond classical histology. Nat. Rev. Cancer.

[16] Eckelmann D., Kusari S., Spiteller M. (2016). Occurrence and spatial distribution of maytansinoids in *Putterlickia pyracantha*, an unexplored resource of anticancer compounds. Fitoterapia.

[17] Beck S., Stengel J. (2016). Mass spectrometric imaging of flavonoid glycosides and biflavonoids in *Ginkgo biloba* L.. Phytochemistry.

[18] Li B., Bhandari D.R., Römpp A., Spengler B. (2016). High-resolution MALDI mass spectrometry imaging of gallotannins and monoterpeneglucosides in the root of *Paeonia lactiflora*. Sci. Rep..

[19] Crecelius A.C., Hölscher D., Hoffmann T., Schneider B., Fischer T.C., Hanke M.V., Flachowsky H., Schwab W., Schubert U.S. (2017). Spatial and temporal localization of flavonoid metabolites in strawberry fruit (*Fragaria* × *ananassa*). J. Agric. Food Chem..

[20] Zhou Q., Wu W.W., Yu C.L., Wang P., Wen X.Q., Chen B.L., Zhang Y., Zhuang M., Zhang M.Y., Zhang H.Y. (2022). Saikosaponin A inhibits growth of human bladder carcinoma T24 and 5637 cells both in vitro and in vivo. Biol. Pharm. Bull..

[21] Zhao H., Wang X., Zhang Y., Huang X., Jiang Y., Ma H., An L., Wu X., Wang Q. (2021). Quantitative ^1^H NMR for the direct Quantification of saikosaponins in *Bupleurum chinense* DC. Anal. Sci..

[22] Xia Z.D., Liu X., Tong L.G., Wang H., Feng M.L., Xi X.H., He P., Qin X.M. (2021). Comparison of chemical constituents of *Bupleurum marginatum* var. *stenophyllum* and *Bupleurum chinense* DC. using UHPLC-Q-TOF-MS based on a metabonomics approach. Biomed. Chromatogr..

[23] Cheng T., Ying M. (2021). Antitumor effect of saikosaponin A on human neuroblastoma cells. Biomed Res. Int..

[24] Lim S.H., Lee H.S., Han H.K., Choi C.I. (2021). Saikosaponin A and D inhibit adipogenesis via the AMPK and MAPK signaling pathways in 3T3-L1 adipocytes. Int. J. Mol. Sci..

[25] Luo J., Wang J., Yang J., Huang W.J., Liu J.Q., Tan W.F., Xin H. (2022). Saikosaponin B1 and Saikosaponin D inhibit tumor growth in medulloblastoma allograft mice via inhibiting the Hedgehog signaling pathway. J. Nat. Med..

[26] Lee J.E., Song B.K., Kim J.H., Siddiqi M.Z., Im W.T. (2022). Production of prosaikogenin F, prosaikogenin G, markogenin F and saikogenin G by the recombinant enzymatic hydrolysis of saikosaponin and their anti-cancer effect. Molecules.

[27] Zhang Q., Li M., Chen X., Liu G., Zhang Z., Tan Q., Hu Y., Fan Y., Liu Y., Zhu T. (2022). Chromosome-Level genome assembly of *Bupleurum chinense* DC provides insights into the saikosaponin biosynthesis. Front. Genet..

[28] Han W., Xu J., Wan H., Zhou L., Wu B., Gao J., Guo X., Sui C., Wei J. (2022). Overexpression of *BcERF3* increases the biosynthesis of saikosaponins in *Bupleurum chinense*. FEBS Open Bio..

[29] Liu W.X., Cheng X.L., Kang R., Wang Y.D., Guo X.H., Jing W.G., Wei F., Ma S.C. (2021). Systematic characterization and identification of saikosaponins in extracts from *Bupleurum marginatum var. stenophyllum* using UPLC-PDA-Q/TOF-MS. Front. Chem..

[30] Liang H., Zhao Y.Y., Cui Y.J., Liu Q.X. (2000). Flavonoids from the roots of *Bupleurum chinense* DC. J. Beijing Med. Univ..

