# Supplementary material for: Spatial Mapping of Bioactive Metabolites in the Roots of Three Bupleurum Species by Matrix-Assisted Laser Desorption/Ionization Mass Spectrometry Imaging
Source: Molecules. 2024 Aug 7;29(16):3746. doi: 10.3390/molecules29163746 (PMC11356868; doi:10.3390/molecules29163746)
Supplement: Supplementary file 1 [file molecules-29-03746-s001.zip › molecules-3110074-supplementary.pdf]

Supplementary Materials

Spatial Mapping of Bioactive Metabolites in the Roots of Three *Bupleurum* Species by Matrix-Assisted Laser Desorption/Ionization Mass Spectrometry Imaging

Xiaowei Han [1,†], Donglai Ma [1,†], Jiemin Wang [1], Lin Pei [2], Lingdi Liu [3], Weihong Shi [1], Zhengpu Rong [1], Xiaoyuan Wang [1], Ye Zhang [1], Yuguang Zheng [1,*] and Huigai Sun [1,*]

[1] College of Pharmacy, Hebei University of Chinese Medicine, Shijiazhuang 050200, China
[2] Hebei Academy of Traditional Chinese Medicine, Shijiazhuang 050031, China
[3] Institute of Cash Crops, Hebei Academy of Agriculture and Forestry Sciences, Shijiazhuang 050051, China
* Correspondence: zyg314@163.com (Y.Z.); sunhuigai66@163.com (H.S.)
† These authors contributed equally to this work.

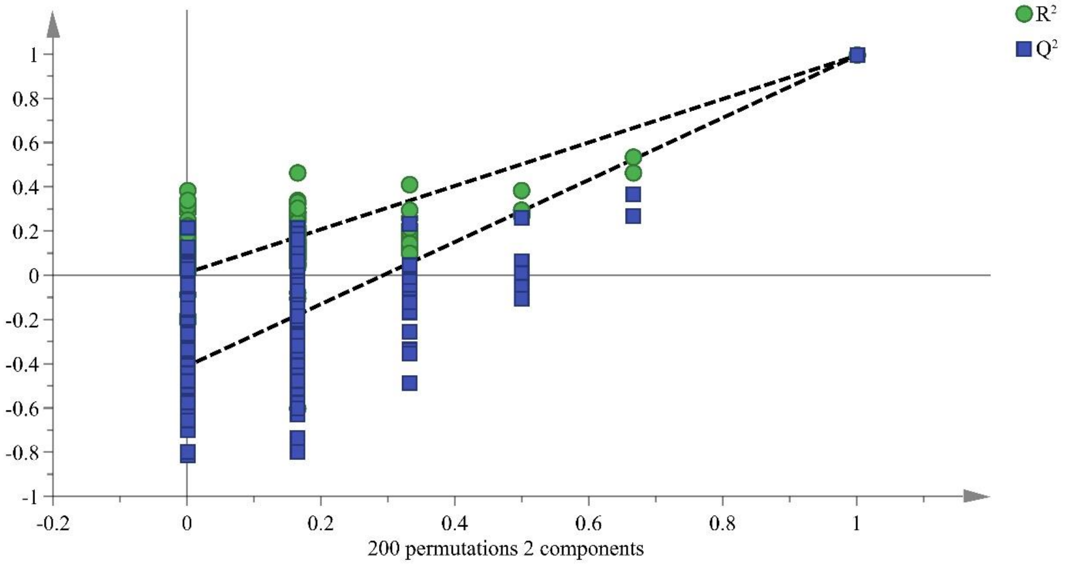

Figure S1. Permutation plot of OPLS-DA.

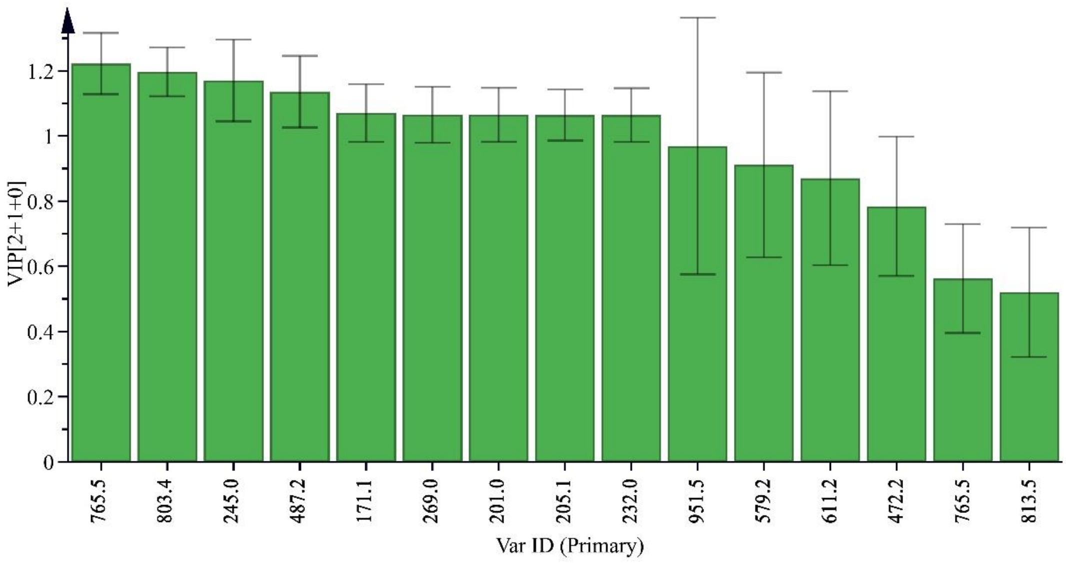

Figure S2. VIP values of saikosaponins, non-saikosaponins, and saikosaponin synthesis pathway compounds in the three Bupleurum species

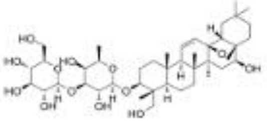

Saikosaponin A

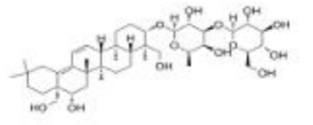

Saikosaponin B1

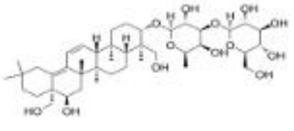

Saikosaponin B2

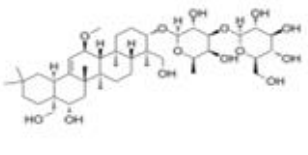

Saikosaponin B3

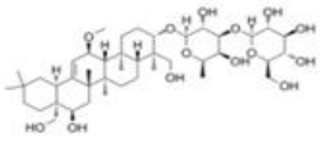

Saikosaponin B4

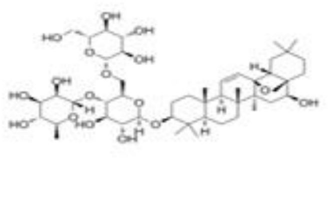

Saikosaponin C

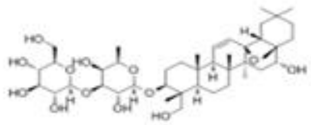

Saikosaponin D

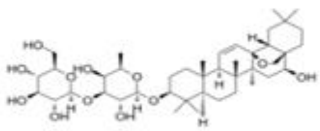

Saikosaponin E

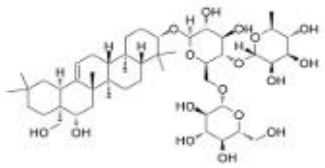

Saikosaponin F

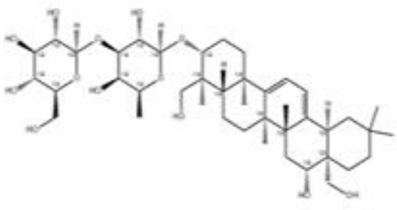

Saikosaponin G

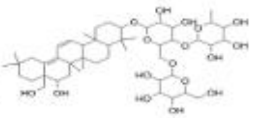

Saikosaponin H

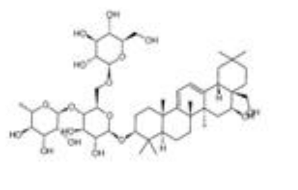

Saikosaponin I

Figure S3. The chemical structure of 12 Saikosaponins in *Bupleurum.*

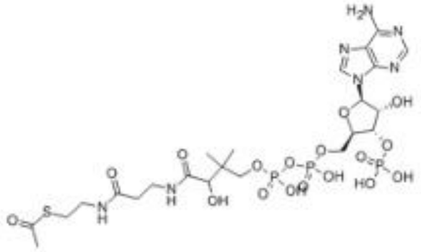

COA

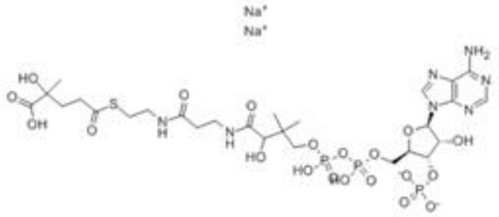

HMG-COA

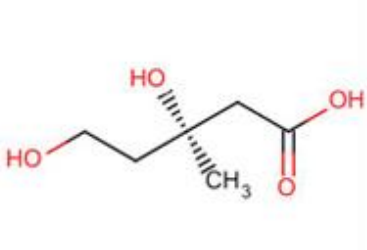

(3R)-3-methylpentanoic acid

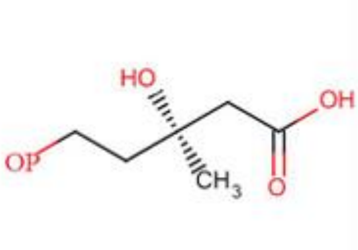

mevalonate-5-phosphate

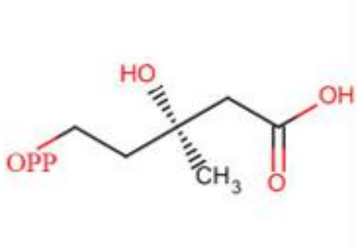

mevalonate-5-pyrophosphate

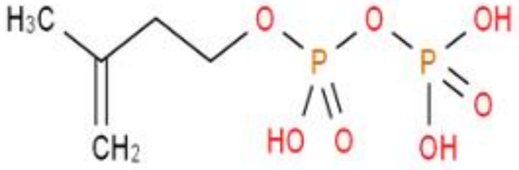

Isopentenyl diphosphate

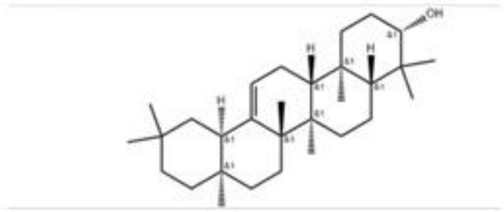

β-amyrin

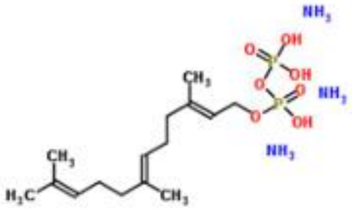

Farnesyl pyrophosphate

Figure S4. The chemical structure of eight compounds of saikosaponin synthesis pathway in *Bupleurum*.

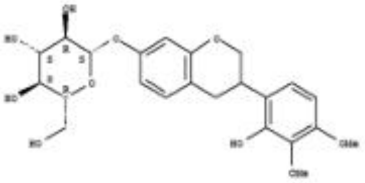

7,2 '- dihydroxy-3', 4 'dimethoxy-isoflavane
-7-o- β- D-glucoside

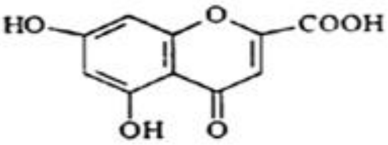

Saikochromone acid

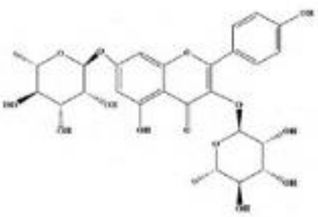

Kaempferitrin

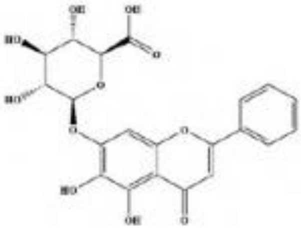

Baicalin

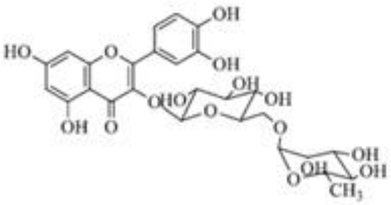

Rutinum

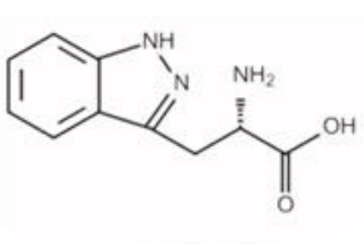

Tryptophan

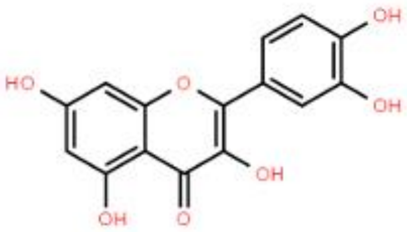

Quercetin

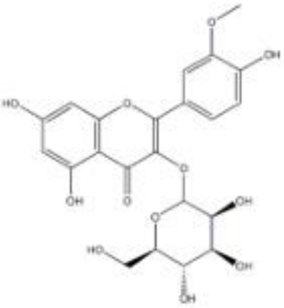

Isorhamnetin-3-O-glucoside

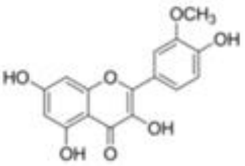

Isorhamnetin

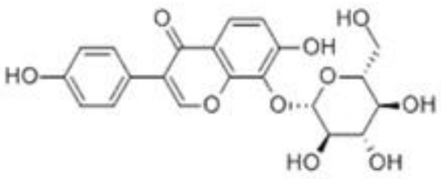

Puerarin

Figure S5. The chemical structure of non-saikosaponin in *Bupleurum*.

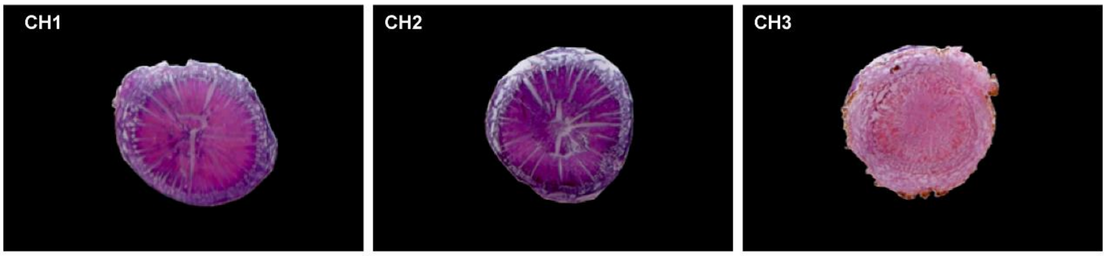

Figure S6. HE staining of three *Bupleurum*. CH1: *Bupleurum smithii*; CH2: *Bupleurum marginatum* var. *stenophyllum*; CH3: *Bupleurum chinense.*

Determination of saikosaponin in three *Bupleurum* species

1. Instruments and materials

Agilent 1260 High Performance Liquid Chromatograph, Agilent 1260 Evaporative Light Scattering Detector (Agilent, USA). Saikosaponin A (content mass fraction ≥98%, lot number A14GB145174), saikosaponin B1 (content mass fraction ≥98%, lot number A22GB146239), saikosaponin B2 (content mass fraction ≥98%, lot number M05GB140794), saikosaponin B3 (content mass fraction ≥98%, lot number M17S11S125048), saikosaponin C (content mass fraction ≥98%, lot number M12O11S126866), saikosaponin D (content mass fraction ≥98%, batch number P27J6F1848), saikosaponin E (content mass fraction ≥98%, batch number M28GB150097), saikosaponin F (content mass fraction ≥98%, batch number M28O10S101347), saikosaponin G (content mass fraction ≥ 98%, batch number M17J11S118582) (Shanghai Yuanye Biotechnology Co., Ltd.). Acetonitrile was chromatographically pure, other reagents were analytically pure, and water was ultrapure water.

2. Chromatographic color parts

The chromatographic column: InertSustain C18 (4.6 mm × 250 mm, 5 μm); the mobile phase: acetonitrile (A) –0.1% formic acid aqueous solution, (B) elution gradient (0~5 min, 35% A; 5~23 min, 35%~42% A; 23~28 min, 42% A; 28~60 min, 42%~48% A; the flow rate: 1.00 mL·min^{-1}, ELSD drift tube temperature: 70 °C; Gas: air; Gas flow rate: 2.3 L·min^{-1};

column temperature: 30 °C.

3. Solution preparation

3.1 Reference solution

Precision weighing of saikosaponin A, B1, B2, B3, C, D, E, F, and G; 1.11 mg, 0.96 mg, 1.29 mg, 1.33 mg, 1.07 mg, 0.69 mg, 0.95 mg, 0.91 mg, and 1.10 mg respectively in nine 5mL volumetric flasks with methanol dissolved and fixed.

3.2 Test solution

Precision weigh 1 g of each of three kinds of Bupleuri radix powder (through 40 mesh sieve), add 50% methanol and 10 mL ultrasonic (500 W 40 Hz) to extract for 45 min, centrifuge 3800 r·min^{-1} for 10 min, and pour out the supernatant. The residue continued to be extracted by 10 mL 50% methanol and ultrasound for 45 min, the supernatant residue was poured out, centrifuged, washed with 10 mL of 50% methanol three times, and combined with the washing solution to recover the solvent under reduced pressure at 50 °C to near dry, and the volume was dissolved and transferred to a 10 mL measuring flask, and filtered with a 0.45 μm microporous filter membrane.
